# Biocontrol Microbial Inoculants Suppress *Fusarium oxysporum*-Associated Disease Symptoms in Rice and Reshape Multicompartment Microbiomes

**DOI:** 10.3390/plants15131986

**Published:** 2026-06-26

**Authors:** Assemgul K. Sadvakasova, Dilnaz E. Zaletova, Meruyert O. Bauenova, Bekzhan D. Kossalbayev, Tao Xu, Dariga K. Kirbayeva, Lazzat Asylbekkyzy, Huma Balouch, Dauren Botbayev, Altynbek A. Abseyt

**Affiliations:** 1 Department of Biotechnology, Faculty of Biology and Biotechnology, Farabi University, Al-Farabi 71, Almaty 050038, Kazakhstan; 2 Department of Chemical and Biochemical Engineering, Institute of Geology and Oil-Gas Business Institute Named After K. Turyssov, Satbayev University, Satpaev 22, Almaty 050043, Kazakhstan; 3 Ecology Research Institute, Khoja Akhmet Yassawi International Kazakh-Turkish University, Turkistan 161200, Kazakhstan; 4 College of Energy and Mining Engineering, Xi’an University of Science and Technology, Xi’an 710054, China

**Keywords:** biological control, rice microbiome, *Fusarium* suppression, cyanobacterial–bacterial inoculants, soil–root–shoot microbiome, community assembly, bacterial-fungal profiling

## Abstract

*Fusarium oxysporum*-associated disease symptoms in rice (*Oryza sativa* L.) seedlings represent an experimentally tractable model for evaluating microbiome-mediated disease suppression under controlled conditions. Biological control of *Fusarium*-associated disease development in rice provides a promising ecological alternative to chemical fungicides. However, the mechanisms underlying the spatial reconfiguration of the host plant multicompartment microbiome in response to complex inoculants remain insufficiently understood. In this study, we investigated the ability of the monoculture *Bacillus amyloliquefaciens* Bn1 (*B. amyloliquefaciens* Bn) and phototrophic–heterotrophic consortia composed of *Nostoc* sp. J-1 and *B. amyloliquefaciens* Bn1 to suppress *Fusarium oxysporum* infection, with parallel profiling of bacterial and fungal communities in rhizosphere soil, the root endosphere, and the phyllosphere using 16S rRNA and ITS amplicon sequencing. Phenotypic screening showed that microbial inoculant application significantly reduced the disease index by up to 55% while maintaining plant dry weight. The protective phenotype was not primarily associated with shifts in alpha diversity, but rather with compartment-specific reorganization of microbial communities. These findings suggest that biological control efficacy was associated less with the overall taxonomic scale of microbiome disturbance than with the formation of a functionally balanced, compartment-specific holobiont architecture but by the formation of a functionally balanced, compartment-specific holobiont architecture, providing a conceptual basis for the targeted design of next-generation phototrophic–heterotrophic biopreparations.

## 1. Introduction

Rice (*Oryza sativa* L.) is among the world’s most important food crops and provides a substantial proportion of dietary calories for more than half of the global population [1]. Under current conditions, the development of rice production is determined not only by the need to increase yield, but also by the requirement to maintain stable production while reducing environmental pressure, using water and soil resources rationally, and limiting dependence on intensive agrochemical inputs [2]. This requirement is particularly relevant for crop protection, because intensive use of chemical fungicides raises substantial concerns for sustainable agriculture due to potential adverse effects on terrestrial, aquatic, and soil organisms [3].

In this context, plant resistance to biotic stressors is therefore a key component of sustainable rice production, because it largely determines the maintenance of crop productivity in sustainable agricultural systems [4]. Among the factors limiting rice productivity, phytopathogenic fungi play a major role, as they can cause pronounced disturbances in plant growth and development and establish persistent infection foci in agrocenoses [5]. For rice, representatives of the genus *Fusarium* are of particular importance because they are associated with damage to seedlings and young plants, persistence of infectious propagules in soil and seed reservoirs, and substantial variability in disease symptoms [6]. This complicates infection control both under field conditions and in model systems, particularly in environmentally heterogeneous agroecosystems, where the outcome of interactions among the plant, pathogen, and associated microbiota is determined by a combination of abiotic and biotic factors [7]. Therefore, effective limitation of *Fusarium*-associated diseases requires not only direct pathogen suppression, but also stabilization of the plant–microbial system.

The limitations of conventional chemical protection become especially evident under variable environmental conditions. The efficacy of fungicide treatments may vary depending on temperature, humidity, soil properties, and the characteristics of the local microbial environment [6]. In addition, repeated application of fungicides imposes selective pressure on pathogen populations and may be accompanied by reduced sensitivity to specific active ingredients [8]. For some representatives of *Fusarium*, including *Fusarium fujikuroi*, resistance to prochloraz has been reported, indicating the limitations of approaches based on the long-term use of a narrow range of chemical protection agents [9]. Chemical pesticides used as antifungal agents may also contribute to contamination of water, soil, and agricultural products, whereas biological control based on plant growth-promoting rhizobacteria has attracted increasing attention because such microorganisms can contribute to soil health, plant growth, and suppression of fungal pathogens [10].

The modern interpretation of biological control is increasingly linked to the concept of the plant as a holobiont, whose functional state is shaped with the participation of associated microbial communities [11,12]. At the same time, the plant microbiome has a pronounced spatial organization. Soil, roots, and leaves differ in physicochemical conditions, substrate availability, and the intensity of host-mediated selection; therefore, the response of microbial communities to pathogen pressure and inoculant application may differ substantially among compartments [13,14]. From this perspective, evaluating the biocontrol effect solely by plant morphometric traits or by the microbiome of a single compartment is insufficient. A more informative approach is the parallel analysis of the bacterial and fungal microbiomes of soil, roots, and leaves in relation to disease intensity and plant growth response [15].

The most extensively studied microbial biocontrol agents include representatives of the genera *Bacillus* and *Pseudomonas*, for which antifungal activity, competition for nutrients and colonization sites, biofilm formation, and the capacity to influence host defense-related signaling have been described in different plant–pathogen systems [16,17]. However, the efficacy of microbial treatment in the plant–soil system is determined not only by the antagonistic potential of an individual strain. Equally important are its ability to establish in appropriate ecological niches, interact with the resident microbiota, and maintain functional activity in a spatially heterogeneous environment [18,19]. For this reason, interest has increased in recent years in different formats of microbial inoculants, including both single-strain preparations and multi-strain compositions, such as synthetic microbial communities that combine organisms with complementary ecological and functional traits [20,21]. In this broader context, biofertilizers composed of beneficial microorganisms, including bacteria and fungi, are increasingly considered economically feasible and environmentally compatible tools with antifungal potential compared with conventional fungicide-centered disease control [22].

In rice agroecosystems, phototrophic microorganisms, particularly cyanobacteria of the genera *Nostoc*, *Anabaena*, and *Trichormus*, are of particular interest. Unlike exclusively heterotrophic inoculants, these organisms can participate in primary production, nitrogen metabolism, and the formation of an extracellular polysaccharide matrix, making them functionally significant components of rhizosphere microbial associations [23,24,25]. Their practical potential is commonly associated with atmospheric nitrogen fixation, exopolysaccharide synthesis, and the production of biologically active compounds, including substances with growth-regulating properties [26,27]. In addition, antimicrobial effects and positive influences on plant physiological status under stress conditions have been described for individual cyanobacterial strains [28,29]. Therefore, cyanobacteria may be regarded not only as a biofertilizer component, but also as phototrophic modules of biocontrol consortia that may support heterotrophic partners and contribute to plant resilience within the pathogen–plant–microbiome system [30,31,32].

Despite considerable progress in the development of microbial biological control agents, it remains insufficiently clear how their protective effect is linked to the reconfiguration of the plant-associated microbiome under *F. oxysporum* infection pressure. In the present study, biological control of *F. oxysporum*-associated disease development in rice is considered as the outcome of interactions among the plant, pathogen, and associated microbiota. This study was designed to compare disease intensity and plant growth status with changes in the bacterial and fungal microbiomes across key compartments of the system. This approach enables assessment of whether reduced disease is accompanied by the formation of compartment-specific microbial signatures and the extent to which these changes are associated with a transition of the plant–soil system toward a more suppressive state.

## 2. Results

### 2.1. Efficacy of Selected Microbial Treatments in Suppressing F. oxysporum-Associated Disease Symptoms and Regulating Rice Growth

The results of the primary in vitro screening showed that the strongest antifungal activity against *Fusarium oxysporum* F679 was observed for the monoculture *B. amyloliquefaciens* Bn1 and several microbial combinations, including *Nostoc* sp. J-1 + *Trichormus variabilis* K-31; *Pseudomonas fluorescens* Un1 + *Bacillus methylotrophicus* Un2 (*B. methylotrophicus* Un2) + *B. amyloliquefaciens* Bn1; *Nostoc* sp. J-1 + *B. amyloliquefaciens* Bn1; *Nostoc* sp. J-1 + *Pseudomonas fluorescens* Un1 + *B. methylotrophicus* Un2 + Bn1; and *Nostoc* sp. J-1 + *Trichormus variabilis* K-31 + *Pseudomonas fluorescens* Un1 + *B. methylotrophicus* Un2 + *B. amyloliquefaciens* Bn1 (Table S1). The highest inhibition of *F. oxysporum* F679 growth was recorded for the binary composition *Nostoc* sp. J-1 + *B.amyloliquefaciens* Bn1, indicating that this phototrophic–heterotrophic combination had the strongest antagonistic effect among the tested variants. Therefore, the most active single-strain and consortium variants were selected for subsequent pot evaluation. In addition, formulations with different volumetric ratios of *Nostoc* sp. J-1 and *B. amyloliquefaciens* Bn1 were included to determine whether the protective response depended on the relative contribution of the phototrophic or heterotrophic component.

**Figure 1:**
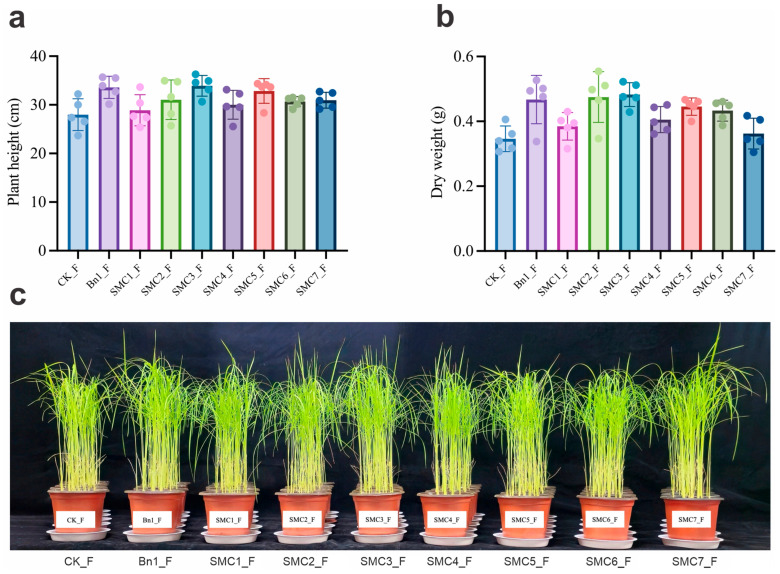
Effects of microbial treatments on plant growth under *F. oxysporum* infection pressure. (**a**) Plant height, (**b**) plant dry weight, and (**c**) rice pot experiments. Data are presented as the mean ± standard deviation (SD) (n = 5). CK_F, pathogen-inoculated control without microbial treatment; Bn1_F, pathogen-inoculated plants treated with *B. amyloliquefaciens* Bn1; SMC1_F–SMC7_F, pathogen-inoculated plants treated with the corresponding microbial consortium variants.

At the next stage, the selected variants were tested in a pot experiment to assess the extent to which the in vitro antifungal activity translated into disease suppression during rice cultivation under *F. oxysporum* infection pressure and whether it was accompanied by changes in plant growth and phytopathological parameters (Figure 1). All groups included in this experiment were challenged with *F. oxysporum* F679; therefore, the suffix “_F” indicates the pathogen-challenged background. For this purpose, the pathogen-inoculated control without microbial treatment (CK_F) was compared with pathogen-inoculated plants treated with *B. amyloliquefaciens Bn1* alone (Bn1_F) and with 7 microbial consortium variants (SMC1_F–SMC7_F).

All microbial inoculation variants were associated with changes in plant growth characteristics relative to CK_F, although the magnitude of this effect varied among treatments. Plant dry weight was generally higher in the inoculated variants than in CK_F (Figure 1b). Relative to CK_F, dry weight increased by approximately 5–39%, depending on the applied treatment. For several consortia, as well as for the single-strain variant, the increase in dry weight exceeded 30%, whereas other consortia showed a more moderate increase, indicating differential effects of microbial community composition on biomass accumulation. Plant height showed lower variability among treatments than dry weight (Figure 1a). Relative to CK_F, the increase in plant height was generally limited and reached approximately 3–7% in individual variants, whereas values in some consortia were comparable to CK_F or slightly lower. Overall, plant height appeared to be a less sensitive indicator of microbial inoculation than biomass-related parameters. A visual assessment of the plant phenotype (Figure 1c) was consistent with the quantitative data, indicating an improvement in the visual condition of the inoculated variants.

**Figure 2:**
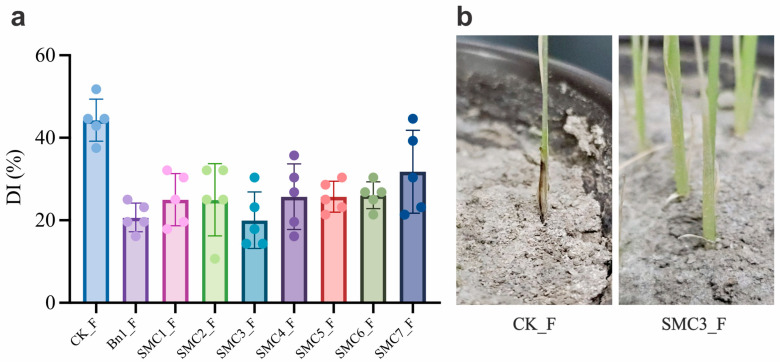
Effects of microbial treatments on disease development under *F. oxysporum* infection pressure. (**a**) Plant disease index under different microbial treatments and (**b**) representative visual symptoms of plants in selected variants. Data are presented as the mean ± standard deviation (SD) (n = 5). CK_F, pathogen-inoculated control without microbial treatment; Bn1_F, pathogen-inoculated plants treated with *B. amyloliquefaciens* Bn1; SMC1_F–SMC7_F, pathogen-inoculated plants treated with the corresponding microbial consortium variants.

The disease index (Figure 2) differed substantially among treatment variants. Compared with CK_F, microbial inoculation reduced the disease index by approximately 28–55%, depending on the variant. The most pronounced reductions were observed under SMC3_F treatment, approximately 55%, and Bn-1_F treatment, approximately 53%, whereas the other consortia reduced the disease index by approximately 30–45%. In all inoculated variants, disease index values were lower than in the CK_F.

Although SMC3_F produced the greatest numerical reduction in disease index, the phenotypic responses of SMC3_F and Bn1_F were closely comparable across the evaluated traits. Therefore, the subsequent microbiome analysis was not based on the assumption that SynCom treatments were uniformly superior to the single-strain treatment. Instead, four groups were retained for microbiome profiling: CK_F, Bn1_F, SMC3_F, and SMC5_F. CK_F represented the pathogen-inoculated control without microbial treatment, whereas Bn1_F represented the single-strain *B. amyloliquefaciens* Bn1 treatment. SMC3_F and SMC5_F were included as phototrophic–heterotrophic consortia with contrasting inoculant architectures, corresponding to a *Bacillus*-dominant and a *Nostoc*-dominant formulation, respectively. This design allowed microbiome-associated biocontrol responses to be evaluated under the same pathogen-inoculated conditions and enabled assessment of whether disease attenuation was linked to distinct bacterial and fungal community reorganization patterns across the soil–root–leaf system.

### 2.2. Diversity and Structure of Bacterial and Fungal Communities

To evaluate the effects of treatments on microbial community structure, bacterial and fungal microbiota profiles were generated based on 16S rRNA and ITS marker sequencing, respectively, in soil, root, and leaf samples. For analysis, the experimental samples were grouped and designated as follows: soil samples (S), CK_F_S, Bn1_F_S, SMC3_F_S, and SMC5_F_S; root samples (R), CK_F_R, Bn1_F_R, SMC3_F_R, and SMC5_F_R; and leaf samples (L), CK_F_L, Bn1_F_L, SMC3_F_L, and SMC5_F_L. In all sample codes, CK_F denotes the pathogen-inoculated control without microbial treatment, whereas Bn1_F, SMC3_F, and SMC5_F denote pathogen-inoculated plants treated with the corresponding microbial inoculants; the final letter indicates the analyzed compartment.

For soil samples based on 16S data, taxonomic annotation covered 43 phyla, 135 classes, 319 orders, 514 families, 1019 genera, and 2092 species, with a total of 7526 OTUs. For soil ITS data, 14 phyla, 47 classes, 111 orders, 232 families, 462 genera, and 653 species were identified, with a total of 2074 OTUs. For roots, 16S profiling identified 28 phyla, 69 classes, 165 orders, 277 families, 532 genera, and 874 species, with a total of 1631 OTUs; ITS profiling identified 10 phyla, 32 classes, 76 orders, 154 families, 264 genera, and 351 species, with a total of 711 OTUs. For leaves, 16S profiling detected 6 phyla, 10 classes, 21 orders, 26 families, 31 genera, and 36 species, with a total of 42 OTUs; ITS profiling detected 7 phyla, 20 classes, 42 orders, 74 families, 101 genera, and 127 species, with a total of 169 OTUs. Overall, the highest taxonomic complexity was observed in soil, followed by roots, whereas the lowest complexity was observed in leaves, particularly for the bacterial component.

Alpha diversity of bacterial and fungal communities was assessed using the ACE richness index and Shannon diversity index across soil, roots, and leaves. The comparison included four pathogen-inoculated groups: CK_F, Bn1_F, SMC3_F, and SMC5_F (Table S2). For both 16S rRNA gene and ITS data, no statistically significant differences in Shannon or ACE indices were detected among treatments in any compartment.

**Figure 3:**
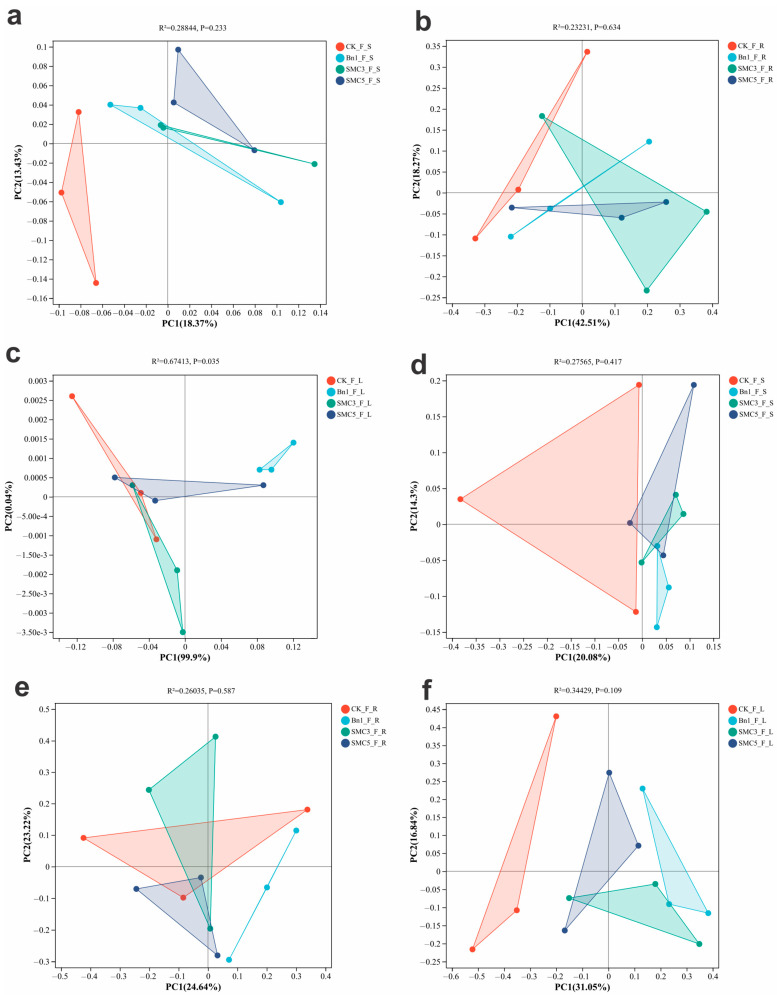
Beta diversity of microbial communities across the soil–root–leaf compartments based on amplicon sequencing. PCoA of bacterial communities (16S rRNA): (**a**) soil (S), (**b**) roots (R), and (**c**) leaves (L). PCoA of fungal communities (ITS): (**d**) soil (S), (**e**) roots (R), and (**f**) leaves (L). CK_F, pathogen-inoculated control without microbial treatment; Bn1_F, pathogen-inoculated plants treated with *B. amyloliquefaciens* Bn1; SMC3_F and SMC5_F, pathogen-inoculated plants treated with the corresponding microbial consortium variants. The final letter in the sample code denotes the compartment: S, rhizosphere soil; R, roots; L, leaves.

Beta diversity was analyzed by PCoA based on Bray–Curtis dissimilarity, with treatment effects assessed by PERMANOVA/Adonis separately for each compartment (Figure 3). For bacterial communities, no significant treatment-associated separation was detected in rhizosphere soil (R^2^ = 0.28844, *p* = 0.233; Figure 3a) or roots (R^2^ = 0.23231, *p* = 0.634; Figure 3b). In contrast, the bacterial community in leaves showed significant intergroup differentiation (R^2^ = 0.67413, *p* = 0.035; Figure 3c). In this compartment, PC1 explained 99.9% of the variation and separated Bn1_F_L and SMC5_F_L from CK_F_L and SMC3_F_L, indicating that treatment-associated bacterial community differences were most clearly detectable in the leaf compartment.

For fungal communities, no statistically significant treatment effect on beta diversity was detected in any compartment. Soil fungal communities did not show significant intergroup separation (R^2^ = 0.27565, *p* = 0.417; Figure 3d), and a similar result was observed for root fungal communities (R^2^ = 0.26035, *p* = 0.587; Figure 3e). In leaves, fungal communities showed partial visual separation among treatments, including separation of CK_F_L from some inoculated variants, but this pattern did not reach statistical significance (R^2^ = 0.34429, *p* = 0.109; Figure 3f).

Overall, microbial inoculation under pathogen-inoculated conditions was not associated with generalized changes in alpha diversity or uniform beta-diversity separation across the soil–root–leaf system. Statistically supported community-level differentiation was restricted to the bacterial microbiota of leaves, whereas bacterial communities in soil and roots and fungal communities in all compartments showed no significant overall separation. These results indicate a selective and compartment-dependent microbiome response to microbial treatments, rather than broad restructuring of the entire bacterial and fungal community.

### 2.3. Compartment-Specific Reconfiguration of Bacterial and Fungal Communities Under Different Treatments at the Genus Level

**Figure 4:**
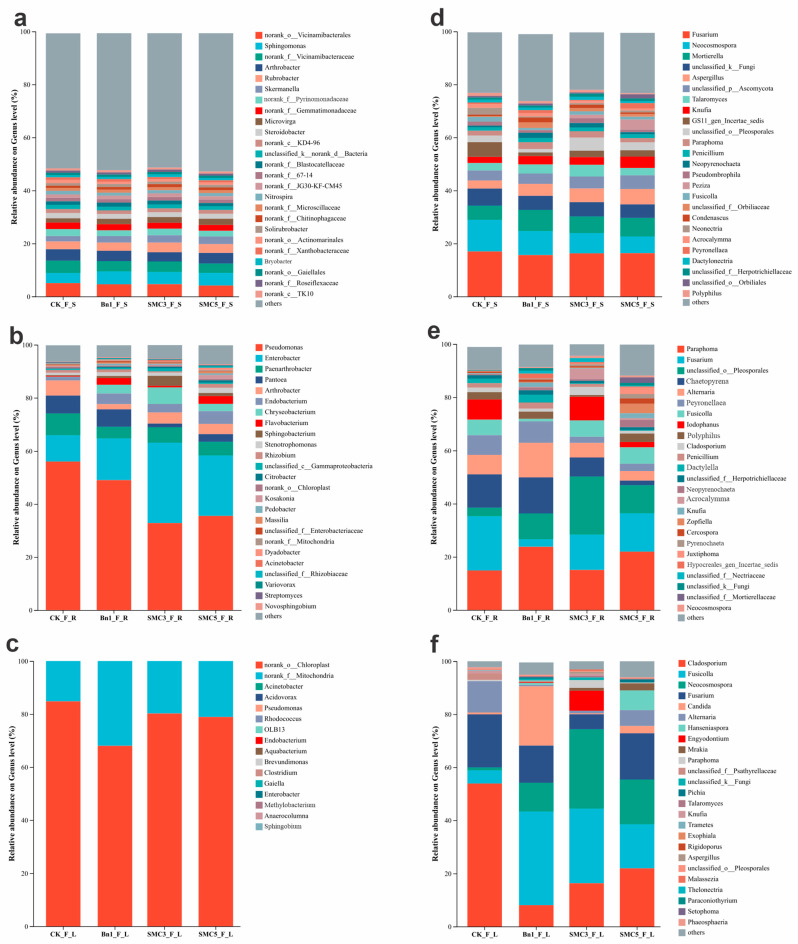
Community composition analysis of bacterial communities (16S rRNA): (**a**) soil (S), (**b**) roots (R), and (**c**) leaves (L), and fungal communities (ITS): (**d**) soil (S), (**e**) roots (R), and (**f**) leaves (L). CK_F, pathogen-inoculated control without microbial treatment; Bn1_F, pathogen-inoculated plants treated with *B. amyloliquefaciens* Bn1; SMC3_F and SMC5_F, pathogen-inoculated plants treated with the corresponding microbial consortium variants. The final letter in the sample code denotes the compartment: S, rhizosphere soil; R, roots; L, leaves.

The taxonomic structure of the microbiome at the genus level was assessed separately for the bacterial component based on 16S rRNA data (Figure 4a–c) and for the fungal component based on ITS data (Figure 4d–f). As all the analysed groups were maintained under conditions of pathogen inoculation, the comparison was focused on treatment-associated differences relative to the pathogen-inoculated control without microbial treatment (CK_F). For each compartment, CK_F was compared with the single-strain treatment Bn1_F and two phototrophic–heterotrophic consortia, SMC3_F and SMC5_F. The Supplementary Materials present detailed percentage values for the dominant genera (Figures S1 and S2).

Based on 16S rRNA gene data, the bacterial community in rhizosphere soil showed a distributed structure without clear hyperdominance of a single genus (Figure 4a). Across CK_F_S, Bn1_F_S, SMC3_F_S, and SMC5_F_S, the dominant and subdominant components included norank_o__Vicinamibacterales, *Sphingomonas*, norank_f__Vicinamibacteraceae, *Arthrobacter*, *Rubrobacter*, and *Skermanella*. Relative to CK_F_S, the inoculated treatments did not show a pronounced shift toward one dominant soil bacterial genus. Instead, the differences among treatments were mainly reflected in moderate redistribution of subdominant taxa. This pattern is consistent with the beta-diversity results, where soil bacterial communities did not show statistically significant overall separation among treatments.

In the root compartment, the main treatment-associated difference was the redistribution of dominant bacterial genera (Figure 4b). CK_F_R was characterized by a strongly *Pseudomonas*-dominated profile, together with visible contributions of *Enterobacter*, *Paenarthrobacter*, *Arthrobacter*, and *Pantoea*. Bn1_F_R retained a broadly similar profile, although the relative contribution of *Pseudomonas* was lower and that of *Enterobacter* was higher than in CK_F_R. In the consortium treatments, this redistribution was more pronounced. SMC3_F_R showed the clearest shift toward an *Enterobacter*-enriched profile, accompanied by a higher relative contribution of *Chryseobacterium* and lower contribution of *Pseudomonas*. SMC5_F_R also showed reduced *Pseudomonas* representation compared with CK_F_R, but retained a more mixed structure, with *Enterobacter*, *Endobacterium*, *Flavobacterium*, and other subdominant genera. Thus, root bacterial differences were mainly reflected in a shift from a *Pseudomonas*-dominated control profile toward more mixed profiles in the inoculated treatments, with SMC3_F_R distinguished by enrichment of *Enterobacter* and *Chryseobacterium* at the relative-abundance level.

In leaves, the 16S rRNA gene profiles were dominated by organelle-assigned categories, mainly norank_o__Chloroplast and norank_f__Mitochondria (Figure 4c). CK_F_L showed the highest relative contribution of the chloroplast-assigned component, whereas Bn1_F_L showed a lower chloroplast-assigned signal and a higher mitochondria-assigned signal. SMC3_F_L and SMC5_F_L occupied intermediate positions. Because the leaf 16S profiles were strongly influenced by organelle-assigned sequences, these data were not used to infer strain-level colonization or persistence of the introduced inoculants in the leaf compartment. Accordingly, the leaf bacterial profile was interpreted conservatively as a treatment-associated amplicon composition pattern rather than as direct evidence of inoculant establishment.

In the ITS profiles, the soil fungal community showed a broadly conserved dominant structure across treatments, with Fusarium, Neocosmospora, Mortierella, unclassified_k__Fungi, *Aspergillus*, and several subdominant ascomycetous lineages representing the main components (Figure 4d). In CK_F_S, Fusarium and Neocosmospora formed prominent fractions of the soil mycobiome. Relative to CK_F_S, the microbial treatments were characterized by a lower relative contribution of Neocosmospora and a higher relative contribution of *Mortierella*. This pattern was observed across the inoculated variants, although the magnitude of the shift differed among Bn1_F_S, SMC3_F_S, and SMC5_F_S. Additional differences among treatments involved moderate redistribution of *Aspergillus*, *Knufia*, GS11_gen_Incertae_sedis, and other low-abundance fungal taxa. Thus, the soil fungal profiles indicate treatment-associated compositional variation within a relatively stable dominant fungal assemblage, with the most consistent pattern being the relative decrease in *Neocosmospora* and the relative increase in *Mortierella* in microbial treatment groups.

In roots, ITS profiles showed more evident treatment-associated differences in the relative structure of dominant fungal genera (Figure 4e). CK_F_R was characterized by substantial contributions of *Paraphoma*, *Fusarium*, *Chaetopyrena*, *Alternaria*, *Peyronellaea*, *Fusicolla*, and *Iodophanus*. Compared with CK_F_R, all microbial treatment groups showed a lower relative contribution of *Fusarium*, although the accompanying redistribution of other dominant genera differed among treatments. Bn1_F_R showed the most pronounced reduction in *Fusarium* representation and was characterized by higher relative contributions of *Paraphoma*, unclassified_o__Pleosporales, *Chaetopyrena*, and *Alternaria*. SMC3_F_R was distinguished by a higher contribution of unclassified_o__Pleosporales, while *Fusarium* remained present at a lower relative level than in CK_F_R. SMC5_F_R also showed a lower relative contribution of *Fusarium* compared with CK_F_R, but retained a mixed fungal profile, with *Paraphoma*, unclassified_o__Pleosporales, *Fusicolla*, and additional subdominant genera contributing to the community structure. Overall, the root ITS data indicate that microbial treatments were associated with redistribution of dominant fungal genera and with a lower relative representation of *Fusarium* compared with the pathogen-inoculated control.

In leaves, the ITS profiles showed clearer treatment-associated differences than those observed in soil (Figure 4f). CK_F_L was characterized by a strongly *Cladosporium*-dominated profile, together with a visible contribution of *Fusarium* and smaller fractions of *Fusicolla*, *Alternaria*, and other genera. Relative to CK_F_L, all inoculated treatments showed lower relative contributions of *Cladosporium* and *Fusarium*, accompanied by a shift toward more multidominant fungal profiles. Bn1_F_L was characterized by increased representation of *Fusicolla* and *Neocosmospora*, together with a distinct contribution of *Candida*. SMC3_F_L showed the most pronounced redistribution toward *Fusicolla* and *Neocosmospora*, with a lower relative contribution of *Fusarium* compared with CK_F_L. SMC5_F_L also showed increased relative contributions of *Fusicolla* and *Neocosmospora*, whereas *Cladosporium* and *Fusarium* were lower than in CK_F_L; this treatment was additionally distinguished by a higher contribution of *Hanseniaspora*. Therefore, the leaf ITS profiles suggest that microbial treatments were associated with a shift away from a strongly *Cladosporium*-dominated mycobiome with detectable *Fusarium* representation toward more evenly distributed fungal assemblages.

### 2.4. Differential Microbiome Structure and Compartment-Specific Candidate Discriminatory Taxa

**Figure 5:**
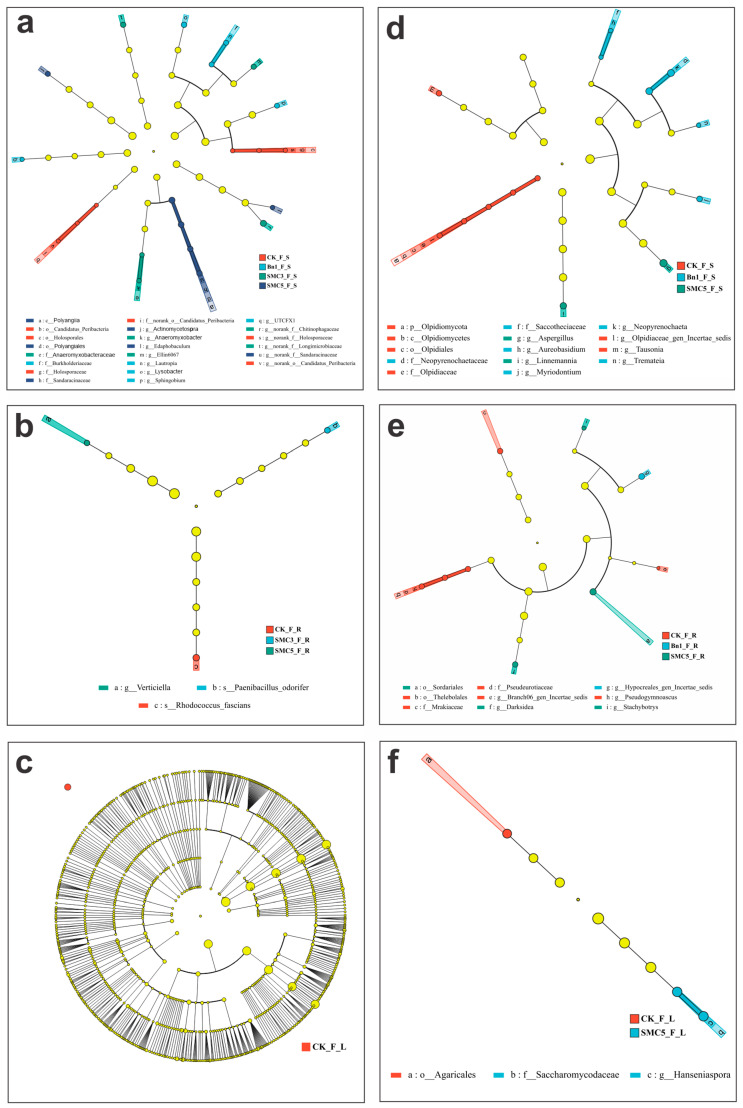
LEfSe analysis of bacterial and fungal microbiomes across soil–root–leaf compartments. Cladograms generated at the threshold log10 LDA > 2.0 and *p* < 0.05 show candidate discriminatory taxa among pathogen-inoculated treatment groups. Panels show bacterial communities based on 16S rRNA gene data in (**a**) soil, (**b**) roots, and (**c**) leaves, and fungal communities based on ITS data in (**d**) soil, (**e**) roots, and (**f**) leaves. Colored nodes indicate candidate discriminatory taxa identified by LEfSe for the corresponding treatment, whereas yellow nodes correspond to taxa that did not reach the LEfSe threshold. CK_F, pathogen-inoculated control without microbial treatment; Bn1_F, pathogen-inoculated plants treated with *B. amyloliquefaciens* Bn1; SMC3_F and SMC5_F, pathogen-inoculated plants treated with the corresponding microbial consortium variants. The final letter in the sample code denotes the compartment: S, rhizosphere soil; R, roots; L, leaves.

The LEfSe analysis was conducted with the objective of identifying candidate discriminatory taxa among the pathogen-inoculated treatment groups: CK_F, Bn1_F, SMC3_F, and SMC5_F. The analysis was conducted separately for bacterial communities based on 16S rRNA gene data and fungal communities based on ITS data across three compartments: rhizosphere soil (S), roots (R), and leaves (L). The LEfSe threshold was set at log10 LDA > 2.0 and *p* < 0.05 (Figure 5).

For bacterial communities, the largest number of candidate discriminatory taxa was detected in rhizosphere soil (Figure 5a). At the genus level, CK_F_S was associated with the unclassified lineage g__norank_f__Holosporaceae and g__norank_o__Candidatus_Peribacteria. Bn1_F_S was associated with *Lautropia*, *Lysobacter*, *Sphingobium*, and g__UTCFX1. SMC3_F_S was associated with *Anaeromyxobacter*, g__Ellin6067, g__norank_f__Chitinophagaceae, and g__norank_f__Longimicrobiaceae. SMC5_F_S was associated with *Actinomycetospora*, *Edaphobaculum*, and g__norank_f__Sandaracinaceae.

In the root bacterial compartment, LEfSe identified a narrow set of discriminatory features (Figure 5b). At the genus level, SMC5_F_R was associated with *Verticiella*. In addition, two species-level markers were detected: *Paenibacillus odorifer* for SMC3_F_R and *Rhodococcus fascians* for CK_F_R. No candidate discriminatory bacterial genus was detected for Bn1_F_R at the applied threshold. Therefore, compared with rhizosphere soil, the root bacterial LEfSe signature was taxonomically more compact and was represented by only a small number of discriminatory features.

In the leaf bacterial compartment, no labelled genus-level bacterial taxon reached the LEfSe threshold (Figure 5c). Accordingly, no candidate discriminatory bacterial genera were assigned to CK_F_L, Bn1_F_L, SMC3_F_L, or SMC5_F_L. This shows that the bacterial community of leaves did not form a reproducible signature of differences among treatments comparable to those observed in soil and roots.

For fungal communities, LEfSe revealed compartment-dependent discriminatory patterns that were more restricted than those observed for the soil bacterial microbiome, but still indicated treatment-associated differentiation in each analyzed compartment (Figure 5d–f). In rhizosphere soil, the LEfSe profile was represented by candidate discriminatory taxa associated mainly with CK_F_S, Bn1_F_S, and SMC5_F_S (Figure 5d). At the genus level, CK_F_S was associated with g__Olpidiaceae_gen_Incertae_sedis and *Tausonia*, whereas Bn1_F_S was associated with *Aureobasidium*, *Myriodontium*, *Neopyrenochaeta*, and *Tremateia*. SMC5_F_S was characterized by *Aspergillus* and *Linnemannia*.

In the root fungal compartment, LEfSe also identified treatment-associated candidate discriminatory taxa, with signatures distributed among CK_F_R, Bn1_F_R, and SMC5_F_R (Figure 5e). At the genus level, CK_F_R was associated with g__Branch06_gen_Incertae_sedis and *Pseudogymnoascus*, while Bn1_F_R was associated with g__Hypocreales_gen_Incertae_sedis. SMC5_F_R was characterized by *Darksidea* and *Stachybotrys*. In addition to these genus-level features, the root fungal profile included higher-rank discriminatory lineages such as o__Sordariales, o__Thelebolales, f__Mrakiaceae, and f__Pseudeurotiaceae.

In the leaf fungal compartment, the LEfSe signature was the most compact among the fungal compartments (Figure 5f). The discriminatory pattern was mainly represented by o__Agaricales for CK_F_L and by f__Saccharomycodaceae together with *Hanseniaspora* for SMC5_F_L. At the genus level, *Hanseniaspora* was the principal candidate discriminatory feature associated with SMC5_F_L. This result is consistent with the community composition analysis at the genus level, where SMC5_F_L showed a distinct contribution of *Hanseniaspora* within a more multidominant leaf fungal assemblage.

### 2.5. Relationships Between the Structure of Bacterial and Fungal Communities in Different Compartments and the Disease Index and Growth Traits Based on Redundancy Analysis (RDA)

Redundancy analysis (RDA) was performed to evaluate the relationships between microbial community structure and plant phenotypic variables under pathogen-inoculated conditions. Bacterial communities were analyzed using 16S rRNA gene data, whereas fungal communities were analyzed using ITS data. The analysis was conducted separately for rhizosphere soil, roots, and leaves and included four pathogen-inoculated groups: CK_F, Bn1_F, SMC3_F, and SMC5_F. Therefore, the ordination patterns were interpreted as associations among microbial treatments under a shared pathogen-inoculated background. Plant disease index (PDI), plant height, and dry weight were fitted as explanatory variables.

**Figure 6:**
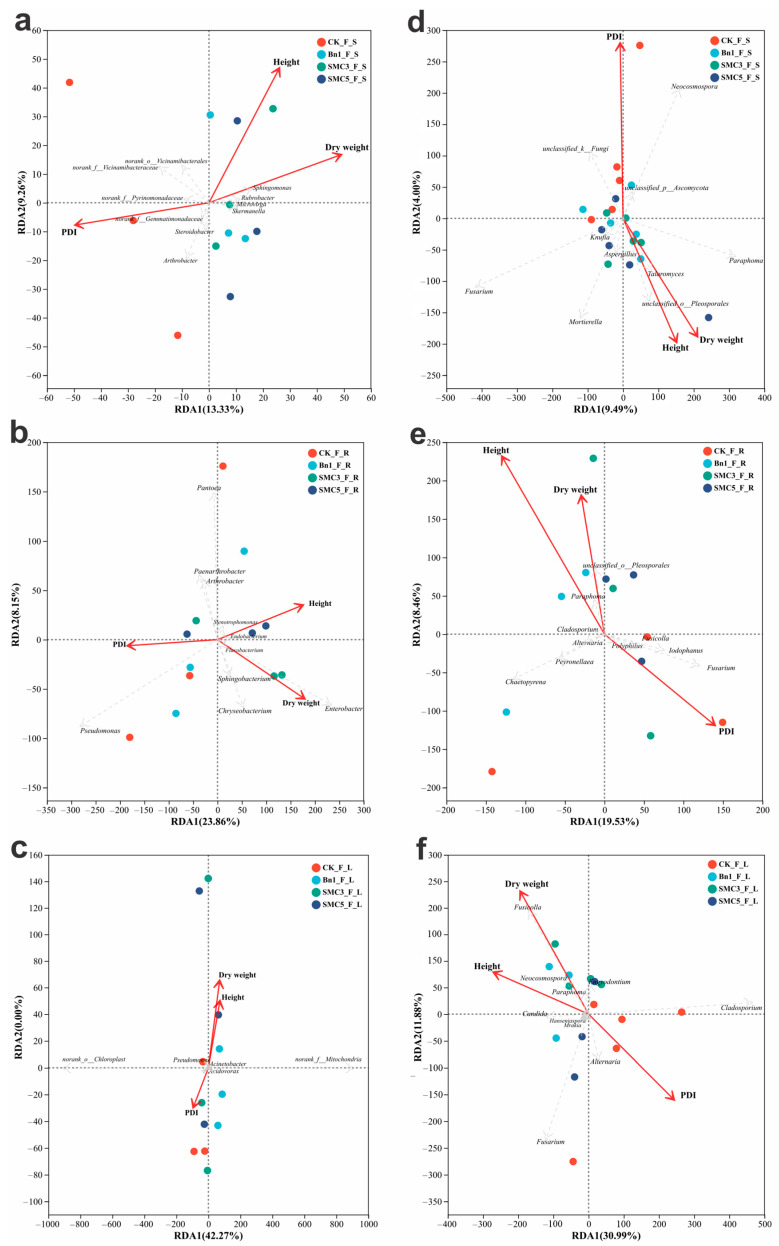
Redundancy analysis (RDA) of bacterial and fungal community structure across soil, root, and leaf compartments under pathogen-inoculated conditions. Panels (**a**–**c**) show RDA ordinations of bacterial communities based on 16S rRNA gene data in soil (**a**), roots (**b**), and leaves (**c**), respectively. Panels (**d**–**f**) show RDA ordinations of fungal communities based on ITS data in soil (**d**), roots (**e**), and leaves (**f**), respectively. Red arrows represent phenotypic variables fitted to community composition, including plant disease index (PDI), plant height, and dry weight. The direction and length of the red vectors indicate the orientation and relative strength of association between phenotypic variables and microbial community structure. Grey dashed arrows represent bacterial or fungal taxa fitted onto the RDA ordination; their direction indicates the samples or treatments with which each taxon is most strongly associated, while their length reflects the relative strength of this association. CK_F, pathogen-inoculated control without microbial treatment; Bn1_F, pathogen-inoculated plants treated with *B. amyloliquefaciens* Bn1; SMC3_F and SMC5_F, pathogen-inoculated plants treated with the corresponding microbial consortium variants. The final letter in the sample code denotes the compartment: S, rhizosphere soil; R, roots; L, leaves.

In rhizosphere soil, RDA of bacterial communities showed that the first two axes explained 22.59% of the variation in community structure, with RDA1 accounting for 13.33% and RDA2 for 9.26% (Figure 6a). The PDI vector was oriented toward the negative side of RDA1, whereas dry weight and height were directed toward the positive ordination space. This configuration indicated an opposite orientation between disease severity and growth-related traits. Most inoculated samples were positioned closer to the positive side of RDA1 than CK_F_S, particularly in the direction of the dry weight and height vectors. Envfit analysis confirmed statistically significant associations between rhizosphere soil bacterial community structure and all three fitted variables: dry weight (r^2^ = 0.60285, *p* = 0.017), height (r^2^ = 0.49746, *p* = 0.047), and PDI (r^2^ = 0.56134, *p* = 0.023). At the genus level, *Sphingomonas*, *Rubrobacter*, *Microvirga*, and *Skermanella* were located closer to the growth-associated side of the ordination, whereas norank_o__Vicinamibacterales, norank_f__Vicinamibacteraceae, norank_f__Gemmatiomonadaceae, and norank_f__Pyrinomonadaceae were positioned closer to the PDI-oriented region. Thus, under pathogen-inoculated conditions, variation in the rhizosphere soil bacterial community was significantly associated with both disease severity and plant growth-related traits.

In roots, bacterial RDA showed that the first two axes explained 32.01% of the variation in community structure, with RDA1 accounting for 23.86% and RDA2 for 8.15% (Figure 6b). The PDI vector was oriented toward the negative side of RDA1, whereas height and dry weight were directed toward the positive side of RDA1. This configuration suggested a separation between disease-associated and growth-associated directions. The *Pseudomonas* vector was positioned in the negative RDA1 region, close to the PDI-oriented side of the plot, whereas *Enterobacter*, *Chryseobacterium*, *Sphingobacterium*, *Endobacterium*, and *Flavobacterium* were located closer to the dry weight and/or height vectors. SMC3_F_R and SMC5_F_R samples were positioned mainly toward the positive RDA1 region, whereas CK_F_R showed broader dispersion, including samples located toward the PDI-associated side. Envfit analysis showed that dry weight was significantly associated with root bacterial community structure (r^2^ = 0.53039, *p* = 0.039). In contrast, height (r^2^ = 0.30361, *p* = 0.189) and PDI (r^2^ = 0.36726, *p* = 0.133) did not reach statistical significance. Therefore, the root bacterial ordination was most strongly supported by its association with dry weight, while the apparent alignment with PDI and height was interpreted as an ordination-based tendency rather than a statistically confirmed relationship.

In leaves, bacterial RDA was dominated by the first canonical axis, with RDA1 explaining 42.27% of the variation and RDA2 explaining 0.00% (Figure 6c). Therefore, interpretation of the leaf bacterial ordination was based mainly on separation along RDA1, and vertical dispersion was not overinterpreted. The PDI vector was directed toward the negative RDA1 and negative RDA2 region, whereas dry weight and height were oriented toward the positive RDA2 direction. The leaf 16S profile was strongly influenced by organelle-assigned features, particularly norank_o__Chloroplast and norank_f__Mitochondria. The bacterial genera *Pseudomonas*, *Acidovorax*, and *Acinetobacter* were positioned close to the center of the ordination. Envfit analysis did not detect statistically significant associations between leaf bacterial community structure and the fitted phenotypic variables: dry weight (r^2^ = 0.33348, *p* = 0.162), height (r^2^ = 0.29320, *p* = 0.198), and PDI (r^2^ = 0.40266, *p* = 0.114). Because the leaf bacterial profile was dominated by organelle-assigned amplicons, RDA2 explained no constrained variation, and envfit associations were not significant, these data were interpreted cautiously as broad compositional patterns rather than as statistically supported bacterial taxon–phenotype relationships.

For the soil fungal community, the first two RDA axes explained 13.49% of the variation in community structure, with RDA1 accounting for 9.49% and RDA2 for 4.00% (Figure 6d). The PDI vector was oriented mainly toward the positive RDA2 direction, whereas dry weight and height were directed toward the positive RDA1 and negative RDA2 region. Envfit analysis indicated that PDI was significantly associated with soil fungal community structure (r^2^ = 0.30823, *p* = 0.031). In contrast, dry weight showed only a non-significant tendency toward association (r^2^ = 0.28033, *p* = 0.067), and height was not significantly associated with the ordination structure (r^2^ = 0.20387, *p* = 0.127). At the genus level, *Neocosmospora* was positioned closer to the PDI-associated region, whereas *Paraphoma*, *Talaromyces*, and unclassified_o__Pleosporales were located closer to the growth-associated direction. *Fusarium* and *Mortierella* were oriented toward the negative RDA1 region.

For the root fungal community, the first two RDA axes explained 27.99% of the variation in community structure, with RDA1 accounting for 19.53% and RDA2 for 8.46% (Figure 6e). The PDI vector was oriented toward the positive RDA1 and negative RDA2 region, whereas dry weight and height were directed toward the opposite sector. Visually, this ordination suggested separation between disease-associated and growth-associated directions in the root mycobiome. However, envfit analysis did not confirm statistically significant associations between root fungal community structure and the measured phenotypic variables: dry weight (r^2^ = 0.14033, *p* = 0.512), height (r^2^ = 0.37958, *p* = 0.105), and PDI (r^2^ = 0.23144, *p* = 0.307). At the genus level, *Fusarium*, *Iodophanus*, *Fusicolla*, and *Polyphilus* were positioned closer to the PDI-oriented sector, whereas *Paraphoma* and unclassified_o__Pleosporales were located closer to the dry weight and height vectors.

For the leaf fungal community, the first two RDA axes explained 42.87% of the variation in community structure, with RDA1 accounting for 30.99% and RDA2 for 11.88% (Figure 6f). The PDI vector was directed toward the positive RDA1 and negative RDA2 region, whereas dry weight and height were oriented toward the negative RDA1 and positive RDA2 region. This opposite orientation indicated that disease severity and growth-related traits were associated with different leaf mycobiome configurations. Envfit analysis confirmed statistically significant associations between leaf fungal community structure and all measured phenotypic variables: dry weight (r^2^ = 0.65688, *p* = 0.003), height (r^2^ = 0.61928, *p* = 0.006), and PDI (r^2^ = 0.63041, *p* = 0.001). At the genus level, *Fusicolla* was strongly oriented toward the dry weight vector. *Cladosporium* was located toward the positive RDA1 region, whereas *Fusarium* was positioned in the negative RDA1 and negative RDA2 sector.

RDA revealed compartment-dependent relationships between microbial community structure and plant phenotypic gradients under pathogen-inoculated conditions. In bacterial communities, ordination patterns in soil and roots suggested an opposite orientation of PDI relative to growth-related traits, whereas the leaf bacterial analysis was constrained by the predominance of organelle-assigned sequences. In fungal communities, the strength of phenotype-associated structure varied among compartments: soil fungal composition was significantly associated with PDI, root fungal composition showed no statistically supported association with the fitted variables, and leaf fungal composition was significantly associated with PDI, dry weight, and height. Therefore, the RDA results support a conservative interpretation in which microbial treatments were associated with compartment-specific microbiome configurations linked to disease and growth gradients, without implying direct causal effects of individual taxa on disease suppression or plant growth.

## 3. Discussion

The present study examined whether microbial inoculants differing in structural and functional complexity, from a single-strain antagonist to phototrophic–heterotrophic synthetic microbial communities, could suppress *F. oxysporum*-associated disease development in rice and reorganize the plant-associated microbiome across soil, root, and leaf compartments. The results demonstrated that selected treatments, notably *B. amyloliquefaciens* Bn1 (Bn1_F) and the SynCom variants SMC3_F and SMC5_F, led to a significant reduction in the plant disease index (PDI) by up to 55%, while maintaining plant dry weight under pathogen pressure. These phenotypic responses were accompanied by compartment-dependent restructuring of bacterial and fungal communities, indicating that the protective phenotype was not limited to a local antagonistic effect against *Fusarium*, but was associated with broader reorganization of the rice holobiont microbiome. Therefore, the results support a microbiome-oriented interpretation of biocontrol, in which disease attenuation may reflect the combined contribution of pathogen limitation, spatial niche restructuring, and host-associated microbial filtering rather than a single inhibitory event [33,34].

Importantly, the SynCom treatments should not be interpreted as uniformly superior to the single-strain Bn1_F treatment at the phenotypic level. The disease index and biomass responses indicated that Bn1_F and SMC3_F produced closely comparable protective outcomes, whereas SMC5_F showed a different balance between disease reduction, plant performance, and microbiome restructuring. Therefore, the added value of the phototrophic–heterotrophic SynComs in this study lies not in a statistically established phenotypic superiority over the single-strain *Bacillus* treatment, but in their distinct ecological mode of action. Bn1_F was associated with a more localized root-protective pattern, whereas the SynCom treatments, particularly SMC3_F and SMC5_F, were associated with broader compartment-dependent reorganization of bacterial and fungal communities across the soil–root–leaf system.

Bn1_F produced the most targeted response at the root interface, where the strongest reduction in the relative abundance of *Fusarium* was observed compared with the pathogen-inoculated control CK_F. This pattern is consistent with the known capacity of *Bacillus* spp. to colonize root-associated niches, form biofilms, compete for nutrients, and produce antifungal lipopeptides capable of inhibiting *Fusarium* growth [35]. However, because strain persistence, root colonization density, and antifungal metabolite production were not directly quantified in this study, the Bn1_F-associated decrease in *Fusarium* should be interpreted as a microbiome-level signature consistent with localized root protection, rather than as direct proof of a specific antagonistic mechanism.

In contrast, the SynCom treatments generated broader microbiome responses, particularly in belowground compartments. The inclusion of a phototrophic cyanobacterial component may alter the ecological context in which heterotrophic antagonists function. Cyanobacteria such as *Nostoc* may contribute fixed nitrogen, extracellular polysaccharides, oxygen, and labile organic compounds, thereby modifying both the resource landscape and the physical structure of rhizosphere microniches [36,37]. In this framework, the phototrophic component should not be viewed only as an additional biofertilizer strain, but as a possible ecological stabilizer that may support heterotrophic partners under fluctuating nutrient conditions [38,39]. At the same time, the contrasting performance of SMC3_F and SMC5_F indicates that increasing the relative contribution of the cyanobacterial module is not sufficient by itself to maximize disease suppression. Rather, SynCom efficacy appears to depend on the balance between rapidly acting heterotrophic antagonism and slower niche-modifying phototrophic functions [40].

The role of *Nostoc* sp. J-1 should therefore be interpreted as part of the designed phototrophic module of the SynCom, not as a strain whose colonization dynamics were directly resolved by the present sequencing approach. Cyanobacteria are amplified by bacterial 16S rRNA primers and may overlap analytically with chloroplast-associated sequences in plant-derived samples; therefore, short-read 16S rRNA profiling cannot provide unambiguous strain-level tracking of the introduced *Nostoc* sp. J-1. This issue is particularly relevant for root and leaf compartments, where plant-derived organelle signals may influence bacterial 16S profiles. Accordingly, the SynCom-associated microbiome shifts observed in this study are interpreted as treatment-level effects of phototrophic–heterotrophic inoculant application, not as direct quantitative evidence of *Nostoc* sp. J-1 persistence or compartment-specific colonization. Direct confirmation of *Nostoc* survival and establishment will require dedicated strain-specific assays.

The differential performance of consortium formulations further indicates that SynCom efficacy depended on functional compatibility rather than inoculant complexity alone. The stronger-performing formulations were those in which *B. amyloliquefaciens* Bn1 was combined with the phototrophic component at ratios that preserved strong root-associated antagonistic potential while adding niche-modifying capacity. This pattern was most evident for SMC3_F, which combined disease suppression with favorable plant performance and microbiome restructuring. In contrast, formulations containing additional heterotrophic or cyanobacterial partners, or a higher relative proportion of the phototrophic module, did not necessarily produce stronger protection. These outcomes may reflect functional redundancy, niche overlap, competition for root-derived substrates, or reduced dominance of the most effective antagonistic member within the consortium. Thus, the lower efficacy of some complex formulations should not be considered a failure of the SynCom concept, but rather evidence that phototrophic–heterotrophic biopreparations require ratio optimization, compatibility-based assembly, and compartmentally aligned microbiome restructuring [41,42,43].

At the level of alpha diversity, the protective phenotype was not associated with a generalized increase in within-sample diversity. Shannon and ACE indices did not show significant treatment-dependent differences for bacterial or fungal communities across rhizosphere soil, roots, or leaves. This result suggests that disease suppression was not driven by a broad increase in taxonomic richness or evenness. Instead, the microbiome response was more consistent with selective compositional rearrangement, compartment-specific enrichment or depletion of particular taxa, and treatment-associated shifts in spatial niche occupancy. This pattern is consistent with the concept that suppressive microbiomes may arise through directional changes in community composition, interaction structure, and functional guild distribution rather than through increased diversity per se [44,45]. Beta-diversity analysis further supported a compartment-dependent, rather than system-wide, microbiome response. Among the six analyzed community–compartment combinations, significant treatment-associated differentiation was detected only for the leaf bacterial community, whereas bacterial communities in rhizosphere soil and roots and fungal communities in all compartments did not show statistically significant treatment-level separation in PCoA. Therefore, the observed protective phenotype should not be interpreted as the result of a broad whole-system beta-diversity shift. Instead, it was associated with more targeted taxonomic and ordination-based signatures, including bacterial restructuring in soil and roots, fungal shifts in soil and leaves, and a particularly strong phenotype-associated configuration of the leaf mycobiome in RDA.

The soil fungal community showed a clear disease-associated restructuring pattern. In the infected control (CK_F_S), *Neocosmospora* was recorded together with *Fusarium*, suggesting the formation of a fungal profile enriched in pathogen-associated or opportunistic taxa. Because *Neocosmospora* is phylogenetically close to fusaria and includes taxa associated with root disease complexes, its increased representation may reflect a broader shift toward a disease-prone fungal assemblage [46]. In the biocontrol-inoculated variants, the relative abundance of *Neocosmospora* decreased, whereas *Mortierella* increased (Figure 4d and Figure S2). Members of *Mortierella* have been frequently associated with plant growth-promoting functions and rhizosphere processes involved in nutrient mobilization, lipid-mediated microbial interactions, and stabilization of soil microbial networks [47,48,49,50]. However, the present data do not demonstrate that *Mortierella* directly suppressed *Fusarium*. A more defensible interpretation is that *Mortierella* enrichment may represent a fungal signature associated with a less disease-prone and more functionally buffered rhizosphere state.

The bacterial community in rhizosphere soil further supported the idea of treatment-specific niche restructuring. In SMC5_F_S, which contained a higher proportion of the cyanobacterial component, increased representation of *Skermanella* and *Microvirga* was observed relative to the infected control CK_F_S. These genera have been associated in previous studies with plant-associated nutrient transformation and, in the case of *Microvirga*, nitrogen-fixing or growth-promoting capacities [51,52]. In addition, *Sphingomonas* was enriched across biocontrol-treated soil samples compared with CK_F_S. This pattern is noteworthy because *Sphingomonas* spp. have been linked to stress tolerance, biofilm formation, and degradation of complex organic compounds, traits that may support persistence in disturbed rhizosphere environments [53]. Nevertheless, the role of these taxa in disease suppression remains inferential. Their enrichment should therefore be interpreted as part of a candidate suppressive bacterial signature, not as direct evidence that these genera caused the reduction in *Fusarium*.

The root endosphere displayed a different pattern. In CK_F_R, the genus *Pseudomonas* showed a marked increase in relative abundance (Figure 4b and Figure S1). This pattern is consistent with a host-mediated ‘cry-for-help’ response, in which pathogen-stressed plants modify root exudation and recruit bacterial groups with potential protective functions [54,55]. *Pseudomonas* spp. are efficient root colonizers and can synthesize diverse antimicrobial metabolites [56]. However, the persistence of a high-disease phenotype in the infected control indicates that this spontaneous recruitment was insufficient to prevent disease development. In the inoculated variants, particularly Bn1_F and SMC3_F, the dominance of *Pseudomonas* decreased, while *Enterobacter* and *Chryseobacterium* became more prominent in the root compartment. Since both genera include strains with reported plant growth-promoting, antioxidant-modulating, and antimicrobial traits [57,58,59], their enrichment may indicate secondary restructuring of the root endosphere under protective treatments. However, this interpretation remains correlative and requires isolation, re-inoculation, and strain-resolved validation.

The leaf compartment provides an additional layer of microbiome-level evidence because microbial treatments were applied to the root zone, whereas fungal community shifts were also detected in leaves. In CK_F_L, the leaf mycobiome was dominated by the opportunistic necrotrophic genus *Cladosporium*, with additional representation of *Alternaria* and *Fusarium*. Such a profile is consistent with colonization of physiologically weakened plant tissues under pathogen stress [60]. Under Bn1_F, SMC3_F and SMC5_F treatments, these pathogen-associated fungal taxa decreased, while *Fusicolla* became more prominent in leaves (Figure 4f and Figure S2). Some representatives of this genus, including *Fusicolla violacea*, have been reported to produce chitinases and β-1,3-glucanases with antifungal potential [61]. Moreover, enrichment of leaf-associated *Fusicolla* has been proposed as a possible marker of a healthy or suppressive belowground microbiome legacy [62]. In the present study, however, enzymatic activity and direct antagonism of the detected *Fusicolla* taxa were not measured. Therefore, *Fusicolla* should be treated as a candidate indicator of phyllosphere restructuring rather than as a confirmed biocontrol agent.

The coordinated response of belowground and aboveground microbial compartments indicates that the response to root-applied inoculants was not confined to the rhizosphere or root endosphere, but was also reflected in the leaf-associated fungal community. This pattern supports the concept of a root–leaf microbiome axis under *F. oxysporum* infection pressure, in which changes in the root-associated microbial environment were associated with downstream restructuring of the phyllosphere mycobiome. A biologically plausible framework for this link is host-mediated chemical filtering: changes initiated at the root interface may influence systemic physiological status and thereby modify the selective environment on leaf surfaces. This interpretation is consistent with previous transcriptomic evidence showing that rice genes involved in phenylpropanoid metabolism, including *OsPAL02*, contribute to phyllosphere microbiome homeostasis through metabolites such as 4-hydroxycinnamic acid [63]. Therefore, the reduction in disease severity together with leaf mycobiome restructuring suggests that biocontrol treatments may be associated not only with local pathogen suppression, but also through broader host–microbiome coordination across plant compartments. Nevertheless, because defense-gene expression, phyllosphere metabolite composition, lignin accumulation, peroxidase activity, and catalase activity were not directly quantified, the present data should be interpreted as an ISR-compatible systemic microbiome pattern rather than as direct experimental evidence of induced systemic resistance.

RDA provided additional support for compartment-dependent relationships between microbiome structure and plant phenotype. In bacterial communities, rhizosphere soil showed statistically significant associations with dry weight, height, and PDI, indicating that soil bacterial composition was aligned with both disease severity and growth-related traits. In roots, bacterial community structure was significantly associated with dry weight, whereas height and PDI did not reach statistical significance. In leaves, bacterial RDA was constrained by the predominance of organelle-assigned sequences and did not show statistically significant associations with the fitted phenotypic variables. For fungal communities, the soil mycobiome was significantly associated with PDI, the root mycobiome showed no statistically supported association with the fitted variables, and the leaf mycobiome was significantly associated with PDI, dry weight, and height. These patterns indicate that disease reduction, plant biomass maintenance, and microbiome restructuring were coordinated but compartment-specific components of the treatment-associated response. Importantly, RDA does not resolve causal direction: microbiome shifts may have contributed to disease attenuation, but they may also have been reinforced by improved plant physiological status.

Functionally, these compartment-specific patterns suggest that the protective response was associated with redistribution of microbial functions across the soil–root–leaf continuum rather than with a uniform increase in microbial diversity. In rhizosphere soil, treatment-associated enrichment of taxa previously linked to nutrient transformation, biofilm-associated persistence, and stress tolerance may indicate a shift toward a more buffered microbial environment under inoculant application. In the root compartment, reduced relative representation of *Fusarium* together with restructuring of dominant bacterial groups suggests a transition of the endosphere toward a community state associated with lower disease severity and improved biomass accumulation. In leaves, treatment-associated mycobiome reorganization indicates that belowground inoculation was reflected in aboveground microbial assembly, potentially through host-mediated filtering processes. Thus, the functional implication of the observed microbiome restructuring is not the replacement of resident microbiota by introduced inoculants, but the redirection of community assembly toward microbial configurations associated with disease attenuation and maintenance of plant performance under *F. oxysporum* infection pressure.

Several methodological limitations should be considered when interpreting these results. First, 16S rRNA and ITS amplicon sequencing are DNA-based approaches and therefore cannot reliably distinguish metabolically active microorganisms from residual extracellular DNA derived from dead or inactive cells. This limitation is particularly relevant for *Fusarium*, because fungal DNA may persist in plant or soil samples even after partial pathogen suppression, potentially leading to overestimation of viable pathogen biomass [64]. Accordingly, changes in the relative representation of *Fusarium* in the present study should be interpreted as DNA-based community signatures rather than as direct measurements of living pathogen abundance. Second, short-read marker-gene sequencing provides limited species- and strain-level resolution, which restricts the ability to distinguish pathogenic, commensal, and beneficial lineages within closely related bacterial or fungal groups [65]. Third, the present study did not directly quantify the persistence of introduced strains, including *Nostoc* sp. J-1, or measure antifungal metabolite production and activation of rice defense pathways. Therefore, the proposed mechanisms should be interpreted as experimentally grounded hypotheses derived from community-level patterns rather than as fully demonstrated causal processes.

Future studies should move from community-level DNA-based associations toward quantitative and functional validation of the proposed biocontrol model. *Fusarium*-specific quantitative assays would help determine whether reduced disease severity corresponds to a decrease in viable pathogen biomass. Strain-specific monitoring of *B. amyloliquefaciens* Bn1, *Nostoc* sp. J-1, and other SynCom members would clarify whether protection is associated with stable inoculant establishment or on transient reorganization of resident microbial communities. In addition, targeted analysis of rice defense-related genes and biochemical markers of phenylpropanoid metabolism and antioxidant defense would be necessary to test whether the observed soil–root–leaf microbiome coordination is accompanied by activation of host defense-related pathways.

The controlled pot design provides a standardized experimental framework characterized by defined pathogen pressure and uniform inoculant application. Accordingly, the observed suppressive microbiome signatures should be interpreted within this controlled context. Field environments introduce additional ecological variability, including heterogeneous soil structure, resident microbial competition, agronomic management, and seasonal effects. Therefore, the stability and agronomic relevance of these signatures require validation in multi-soil and multi-season field trials. Finally, multi-soil and multi-season field trials are required to determine whether the suppressive microbiome signatures observed under controlled pot conditions remain stable under agronomically variable field environments [66,67].

Overall, this study indicates that suppression of *F. oxysporum*-associated disease development in rice by Bn1_F and phototrophic–heterotrophic SynComs was associated with coordinated restructuring of bacterial and fungal communities across rhizosphere soil, roots, and leaves. The main conceptual advance is that SynCom efficacy appears to depend not only on the presence of beneficial microorganisms, but also on the functional balance between heterotrophic antagonists and phototrophic stabilizers. Bn1_F was associated with a more localized root-protective pattern, whereas SynCom treatments induced broader multicompartment microbiome reorganization. These findings provide a basis for microbiome-oriented selection of next-generation biopreparations, while also defining the functional experiments required to move from correlative microbial signatures to experimentally validated mechanisms.

## 4. Materials and Methods

### 4.1. Microorganisms and Culture Maintenance

The experimental microbial pool was formed by integrating photoautotrophic and heterotrophic taxa selected according to criteria of confirmed biocontrol potential and interspecific functional complementarity.

Cyanobacterial strains *Nostoc* sp. J-1 (PZ233676) and *Trichormus variabilis* K-31 (MZ079356) from the collection of the Kazakh-Japanese-Chinese International Research Center Algabiotech at Farabi University were used as the phototrophic component. The heterotrophic component was represented by strains isolated from rhizosphere soils of rice agrocenoses in the Almaty region, Kazakhstan: *Pseudomonas fluorescens* Un1 (PZ230248), *B. methylotrophicus* Un2 (PZ229617), and *B. amyloliquefaciens* Bn1 (PZ229648).

Phototrophic strains were cultivated in mineral BG-11 medium under standard illumination conditions, whereas heterotrophic bacteria were maintained in liquid TSB medium. Long-term storage of cultures was performed at −80 °C in 25% (*v*/*v*) sterile glycerol. Before the experiment, cultures were transferred to fresh nutrient media, and cells in the active growth phase, either exponential or early stationary, were used.

The phytopathogenic agent used in this study was *Fusarium oxysporum* F679 (PV450006), obtained from the collection of the Applied Microbial Ecology Engineering Laboratory, Tianjin Institute of Industrial Biotechnology, Chinese Academy of Sciences. The culture was maintained on potato dextrose agar (PDA) at 4 °C with regular subculturing every 14 days. To obtain a standardized inoculum, the fungus was cultivated on PDA at 28 °C for 7 days until mature mycelium and abundant sporulation were formed.

### 4.2. Preparation and Standardization of Microbial Suspensions

The photoautotrophic strains *Nostoc* sp. J-1 and *Trichormus variabilis* K-31 were grown separately in BG-11 medium at 27 °C in 1 L flasks with a working volume of 500 mL for 7 days under an irradiance of 50 μmol photons m^−2^ s^−1^. Aeration was performed with sterile air passed through a membrane filter with a pore diameter of 0.22 μm. At the end of cultivation, phototrophic biomass was harvested by centrifugation at 5000× *g* for 10 min, the supernatant was removed, and the cell pellet was washed twice with sterile deionized water to remove residual BG-11 culture medium. After washing, the biomass was resuspended in sterile deionized water to obtain a homogeneous suspension and standardized by optical density to OD_720_ = 1.5.

The heterotrophic strains *Pseudomonas fluorescens* Un1, *B. methylotrophicus* Un2, and *B. amyloliquefaciens* Bn1 were grown separately in liquid TSB medium at 30 °C on an orbital shaker at 180 rpm for 18–24 h. Cells were harvested by centrifugation at 5000× *g* for 10 min at 4 °C, washed twice with sterile deionized water, and resuspended in sterile deionized water. The density of bacterial suspensions was adjusted to 1.0 × 10^8^ CFU/mL^−1^.

The inoculum of *Fusarium oxysporum* F679 was prepared by washing conidia from a 7-day-old PDA culture with sterile deionized water containing 0.05% (*v*/*v*) Tween-20. The suspension was filtered through two layers of sterile gauze to remove mycelial fragments and adjusted to 1.0 × 10^6^ conidia/mL^−1^ based on hemocytometer counts. Conidial viability was confirmed using a germination assay and was considered acceptable when germination was not lower than 90% after 12 h of incubation at 28 °C.

### 4.3. Primary Screening of Antagonistic Activity In Vitro

To preliminarily select the most promising microbial compositions, in vitro screening of antagonistic activity against *Fusarium oxysporum* was performed. The assay included live standardized cell suspensions of individual strains and their mixtures, prepared immediately before the experiment from the working inocula described in Section 4.2. The primary screening included 22 variants, comprising single-strain cultures, binary combinations, three-component combinations, four-component mixtures, and the complete five-strain variant.

Before preparing mixed inocula, potential strain compatibility was evaluated by the cross-streak method on agarized nutrient medium. The absence of pronounced growth suppression zones or clear inhibition boundaries in the contact area was considered evidence of the absence of direct interstrain antagonism and served as the basis for further co-testing.

Antagonistic activity was evaluated using the dual-culture agar method. A 5 mm disc of actively growing *F. oxysporum* mycelium was placed in the center of a 90 mm Petri dish containing 20 mL of PDA. A 10 μL suspension of the tested strain or microbial composition was applied to the agar surface at 25 mm from the fungal disc. In the control variant, an equal volume of sterile deionized water was applied instead of the microbial suspension. After incubation at 25 °C for 7 days, radial mycelial growth was measured, and the percentage inhibition was calculated using the formula:

PI (%) = [(Rc − Rt)/Rc] × 100,(1)

where Rc denotes radial pathogen growth in the control variant and Rt denotes radial pathogen growth in the presence of the tested inoculant. All variants were analyzed in three independent biological replicates.

### 4.4. Pot Experiment and Inoculation Scheme

Rice (*Oryza sativa* L.) was used as the model plant. Experiments evaluating the plant growth-promoting activity of the strains were conducted using seeds of the three-line indica rice hybrid Nei 5 You (hybrid 8015; Zhejiang Agricultural Science and Technology Seed Industry Co., Ltd., Huzhou, China).

Seeds were washed with running water, surface-sterilized in 70% ethanol for 2 min and 2% NaOCl for 2 min, followed by five washes with sterile water, and then germinated at 30 °C in darkness for 48 h on moistened sterile filter paper. Germinated seeds were sown at 15 seeds per plastic pot containing 240 g of homogenized soil sieved through a 5 mm mesh. On day 5 after seedling emergence, plant density was standardized by removing weak plants and leaving 12 morphologically uniform seedlings per pot.

The experiment was established using a completely randomized design. Each treatment included five biological replicates (n = 5), and one pot was considered an independent experimental unit. Pots were maintained under a 16 h photoperiod, 70–75% relative humidity, and a temperature regime of 28 °C during the day and 22 °C at night; pot positions were regularly randomized.

Only microbial variants that demonstrated the strongest antifungal activity against *Fusarium oxysporum* F679 during the primary in vitro screening were included in the pot experiment. At the two-leaf stage, the corresponding microbial suspension was applied to each pot by root-zone drenching. The total inoculum volume in all variants was 60 mL per pot. The pathogen-inoculated control without microbial treatment (CK_F) received an equivalent volume of sterile deionized water instead of a microbial suspension (Table 1).

At 24 h after microbial treatment application, all treatment variants, including CK_F, were artificially inoculated with 60 mL of *Fusarium oxysporum* F679 suspension (1.0 × 10^6^ conidia mL^−1^) by root-zone drenching. Thus, CK_F represented the pathogen-inoculated control without microbial treatment, whereas Bn1_F and SMC1_F–SMC7_F represented pathogen-inoculated plants treated with the corresponding microbial preparations. Watering was performed every 1–2 days using exactly 60 mL per pot of settled tap water. The experiment lasted 30 days after pathogen inoculation.

### 4.5. Assessment of Disease Development and Growth Parameters

At 30 days after pathogen inoculation, plants were carefully removed from the soil, roots were washed under running water to remove adhering substrate, and plant material was blotted dry with filter paper. Each treatment consisted of five independent pots, with 12 plants maintained per pot. Morphometric measurements were recorded for all plants within each pot, including shoot height (cm), root length (cm), stem diameter (mm), and fresh weight of shoots and roots (g). For dry weight determination, plant samples were dried at 65 °C to constant weight for 72 h and then weighed. For statistical analysis, the mean value calculated for each pot was used as one independent biological replicate (n = 5).

Disease symptoms were assessed on a 0–4 scale: 0, no symptoms; 1, mild symptoms; 2, moderate symptoms; 3, severe symptoms; and 4, plant death or extremely severe symptoms. The disease index (DI, %) was calculated as:

(2) $$DI\;\left(\%\right)=\frac{\sum \left(s_{i}\times n_{i}\right)}{4\times N}\times 100,$$

where s_i_ is the disease score, n_i_ is the number of plants with score s_i_, and N is the total number of assessed plants.

### 4.6. Sample Collection for Analysis

At the end of the experiment, samples of rhizosphere soil, roots, and leaves were collected. Rhizosphere soil was defined as the soil fraction tightly associated with the roots and detached after gentle shaking of the root system. Roots were washed to remove residual soil and used as root samples. Leaves were collected from the aboveground part of the plants, forming separate samples for analysis of the leaf compartment.

For 16S rRNA and ITS amplicon sequencing, treatment variants that showed the best performance based on phenotypic screening were selected, namely reduced disease index and improved or maintained growth parameters relative to the pathogen-inoculated control CK_F. The selected groups were CK_F, Bn1_F, SMC3_F, and SMC5_F. For each selected treatment, rhizosphere soil, root, and leaf samples were analyzed separately. All samples were immediately frozen and stored at −80 °C until DNA extraction.

### 4.7. DNA Extraction, PCR Amplification, and Illumina Sequencing

Microbial DNA was extracted from rhizosphere soil, root, and leaf samples using the E.Z.N.A.^®^ Soil DNA Kit (Omega Bio-tek, Norcross, GA, USA) according to the manufacturer’s instructions. DNA quality and concentration were assessed by electrophoresis in a 1.0% agarose gel and using a NanoDrop2000 spectrophotometer (Thermo Scientific, Waltham, MA, USA). DNA was stored at −80 °C.

The bacterial community was characterized using the V3–V4 region of the 16S rRNA gene with primers 338F (5′-ACTCCTACGGGAGGCAGCAG-3′) and 806R (5′-GGACTACHVGGGTWTCTAAT-3′). The fungal community was characterized using the ITS2 region with primers ITS1F (5′-CTTGGTCATTTAGAGGAAGTAA-3′) and ITS2R (5′-GCTGCGTTCTTCATCGATGC-3′). Amplification conditions followed the standard protocols of the sequencing provider for the specified primer pairs.

PCR products were excised from a 2% agarose gel, purified using the PCR Clean-Up Kit (YuHua, Shanghai, China), and quantified using a Qubit 4.0 fluorometer (Thermo Fisher Scientific, Waltham, MA, USA). Purified amplicons were pooled in equimolar amounts and subjected to paired-end sequencing on the Illumina NextSeq2000 platform (Illumina, San Diego, CA, USA) according to the standard protocols of Majorbio Bio-Pharm Technology Co., Ltd. (Shanghai, China). Raw data were deposited in the NCBI SRA under accession number PRJNA1448066.

### 4.8. Data Analysis

All quantitative data are presented as the mean ± standard deviation (SD). Plant phenotypic parameters, including the plant disease index (PDI), plant height, and dry weight, were compared among treatments using one-way analysis of variance (one-way ANOVA), followed by multiple comparisons among groups. Differences were considered statistically significant at *p* < 0.05.

Data processing and bioinformatic analysis of the soil, root, and leaf microbiota were performed using the Majorbio Cloud platform (https://cloud.majorbio.com). Raw paired-end reads were assigned to samples according to barcode information and subjected to quality control according to the standard Majorbio workflow. Low-quality reads, sequences containing ambiguous bases, primer or barcode artifacts, and chimeric sequences were removed before downstream analysis. High-quality sequences were clustered into operational taxonomic units (OTUs), and representative sequences were used for taxonomic annotation of bacterial 16S rRNA and fungal ITS datasets. To reduce bias caused by unequal sequencing depth among samples, OTU abundance tables were normalized before diversity analyses. Rarefaction curves were inspected to evaluate whether the sequencing depth was sufficient to represent the dominant bacterial and fungal diversity in each compartment. Based on OTU information, alpha diversity indices were calculated using Mothur v1.30.1 [68]. Similarity among microbial communities in different samples was determined by principal coordinate analysis (PCoA) based on Bray–Curtis dissimilarity using the Vegan v2.5-3 package. The PERMANOVA test was used to assess the percentage of variation explained by treatment together with its statistical significance using the Vegan v2.5-3 package. Linear discriminant analysis effect size (LEfSe) [69] was applied as an exploratory differential-abundance approach to identify candidate discriminatory taxa among treatments from phylum to genus level. Taxa were retained when they satisfied both the statistical significance threshold of *p* < 0.05 and the effect-size threshold of log10 LDA > 2.0. To evaluate the relationship between variation in microbial community structure and plant phenotypic traits, redundancy analysis (RDA) was applied separately to bacterial (16S rRNA) and fungal (ITS) communities in each compartment: soil, roots, and leaves. Plant disease index (PDI), plant height, and dry weight were used as explanatory variables because these traits reflected the main pathological and growth-response gradients in the pot experiment. Associations between fitted variables and microbial community structure were evaluated using a permutation-based envfit procedure, and relationships were considered statistically significant at *p* < 0.05.

## 5. Conclusions

This study showed that biocontrol microbial preparations substantially attenuated *F. oxysporum*-associated disease development in rice seedlings while improving or maintaining plant growth parameters under controlled infection pressure. The strongest protective responses were observed under single-strain inoculation with *B. amyloliquefaciens* Bn1 (Bn1_F) and under treatment with the phototrophic–heterotrophic consortium variants SMC3_F and SMC5_F, as reflected by reduced plant disease index and maintained or increased dry weight relative to the pathogen-inoculated control CK_F. The results indicate that biocontrol efficacy in this system was not associated with complete elimination of the pathogen-associated signal from the rhizosphere environment, but rather with pronounced compartment-specific reconfiguration of bacterial and fungal communities across the soil–root–leaf system. In rhizosphere soil, microbial treatments were associated with restructuring of bacterial and fungal relative-abundance profiles, including shifts in candidate taxa linked to nutrient transformation, stress tolerance, and fungal community buffering. In roots, treatment-associated changes included reduced relative representation of *Fusarium* in the fungal community and reorganization of dominant bacterial genera relative to CK_F. In leaves, treatment-associated restructuring of the mycobiome was also detected, indicating that root-applied inoculants were associated with microbiome responses beyond the site of application. Therefore, the data support a conservative microbiome-oriented interpretation of biocontrol, in which microbial preparations may contribute to disease attenuation not only through local pathogen limitation, but also through broader reorganization of plant-associated microbial communities. At the same time, these patterns should be interpreted as DNA-based community signatures and phenotype-associated microbiome configurations rather than as direct proof of causal mechanisms involving individual taxa, introduced-strain persistence, or induced systemic resistance. Collectively, the findings support the potential of microbial inoculants, including formulations designed with a phototrophic cyanobacterial component, for the development of environmentally safer next-generation biopreparations for rice cultivation. Further strain-specific tracking, pathogen quantification, host-defense assays, and field validation will be required to confirm the mechanisms and agronomic stability of the observed suppressive microbiome signatures.

## 6. Patents

The cyanobacterial strain *Nostoc* sp. J-1 used in this study is disclosed in Kazakhstan patent for invention No. 37641, “Strain of the cyanobacterium *Nostoc* sp. J-1 used to increase crop yields and protect against phytopathogenic fungi” (application No. 2024/0390.1; publication date: 28 November 2025).

## Figures and Tables

**Table 1:** Treatment variants and composition of inoculation mixtures.

Treatment	Components	Volumes (mL)	Component Ratio (*v*/*v*)
CK_F	Sterile deionized water	60	-
Bn1_F	*B. amyloliquefaciens* Bn1	60	Bn1 = 1
SMC1_F	*Nostoc* sp. J-1 + *Trichormus variabilis* K-31	30 + 30	J-1:K-31 = 1:1
SMC2_F	*Pseudomonas fluorescens* Un1 + *B. methylotrophicus* Un2 + *B. amyloliquefaciens* Bn1	20 + 20 + 20	Un1:Un2:Bn1 = 1:1:1
SMC3_F	*B. amyloliquefaciens* Bn1 + *Nostoc* sp. J-1	40 + 20	Bn1:J-1 = 2:1
SMC4_F	*Nostoc* sp. J-1 + *Pseudomonas fluorescens* Un1 + *B. methylotrophicus* Un2 + *B.* *amyloliquefaciens* Bn1	30 + 10 + 10 + 10	J-1:Un1:Un2:Bn1 = 3:1:1:1
SMC5_F	*Nostoc* sp. J-1 + *B.* *amyloliquefaciens* Bn1	40 + 20	J-1:Bn1 = 2:1
SMC6_F	*Nostoc* sp. J-1 + *Trichormus variabilis* K-31 + *B.* *amyloliquefaciens* Bn1	15 + 15 + 30	J-1:K-31:Bn1 = 1:1:2
SMC7_F	*Nostoc* sp. J-1 + *Trichormus variabilis* K-31 + *Pseudomonas fluorescens* Un1 + *B. methylotrophicus* Un2 + *B. amyloliquefaciens* Bn1	12 + 12 + 12 + 12 + 12	J-1:K-31:Un1:Un2:Bn1 = 1:1:1:1:1

## Data Availability

All data generated or analyzed during this study are included in this article and its Supplementary Information files. Amplicon sequencing data supporting the findings of this study are openly available in the NCBI Sequence Read Archive (SRA) under BioProject accession number PRJNA1448066.

## Supplementary Materials

The following supporting information can be downloaded at: https://www.mdpi.com/article/10.3390/plants15131986/s1, Table S1: Primary in vitro screening of antagonistic activity against *Fusarium oxysporum* F679; Table S2: Alpha-diversity indices (Shannon, ACE) across compartments for 16S rRNA and ITS; Figure S1: Relative abundance of dominant bacterial genera under different treatments; Figure S2: Relative abundance of dominant fungal genera under different treatments.

## Abbreviations

The following abbreviations are used in this manuscript:

SynCom	Synthetic Microbial Community
PGPR	Plant Growth-Promoting Rhizobacteria
16S rRNA	16S ribosomal RNA
ITS	Internal Transcribed Spacer
DNA	Deoxyribonucleic Acid
PCR	Polymerase Chain Reaction
OTU	Operational Taxonomic Unit
ASV	Amplicon Sequence Variant
PCoA	Principal Coordinates Analysis
PERMANOVA	Permutational Multivariate Analysis of Variance
LDA	Linear Discriminant Analysis
LEfSe	Linear Discriminant Analysis Effect Size
RDA	Redundancy Analysis
db-RDA	Distance-based Redundancy Analysis
ANOVA	Analysis of Variance
SD	Standard Deviation
PDI	Plant Disease Index
DI	Disease Index
NCBI	National Center for Biotechnology Information
SRA	Sequence Read Archive
TSB	Tryptic Soy Broth
TSA	Tryptic Soy Agar
PDA	Potato Dextrose Agar
OD	Optical Density
CFU	Colony-Forming Units
