# Supplementary material for: Biocontrol Microbial Inoculants Suppress Fusarium oxysporum-Associated Disease Symptoms in Rice and Reshape Multicompartment Microbiomes
Source: Plants (Basel). 2026 Jun 26;15(13):1986. doi: 10.3390/plants15131986 (PMC13364377; doi:10.3390/plants15131986)
Supplement: Supplementary file 1 [file plants-15-01986-s001.zip › Тable S2.pdf]

Table S2. Alpha-diversity indices (Shannon, ACE) across compartments for 16S rRNA and ITS

Marker	Compartment	Group	Shannon (mean ± SD)	ACE (mean ± SD)
16S	Leaf (L)	CK_F_L	0.41944 ± 0.09214 a	1.67 ± 2.89 a
		Bn1_F_L	0.62666 ± 0.01415 a	3.00 ± 2.65 a
		SMC3_F_L	0.49578 ± 0.04565 a	3.52 ± 3.06 a
		SMC5_F_L	0.50206 ± 0.10735 a	7.86 ± 9.05 a
	Root (R)	CK_F_R	1.75342 ± 0.62869 a	252.78733 ± 14.52707 a
		Bn1_F_R	1.93062 ± 0.55616 a	217.94515 ± 55.19682 a
		SMC3_F_R	2.03475 ± 0.24164 a	277.91832 ± 71.43744 a
		SMC5_F_R	2.34807 ± 0.27101 a	293.64064 ± 64.81057 a
	Soil (S)	CK_F_S	4.9965 ± 0.0819 a	705.55 ± 19.89 a
		Bn1_F_S	5.0226 ± 0.0582 a	691.67 ± 16.50 a
		SMC3_F_S	5.0160 ± 0.0653 a	681.83 ± 37.65 a
		SMC5_F_S	5.0485 ± 0.0187 a	668.26 ± 11.27 a
ITS	Leaf (L)	CK_F_L	1.0145 ± 0.8537 a	14.62 ± 3.12 a
		Bn1_F_L	1.4986 ± 0.1801 a	13.00 ± 11.36 a
		SMC3_F_L	1.5883 ± 0.2287 a	0.00 ± 0.00 a
		SMC5_F_L	1.7686 ± 0.3373 a	6.23 ± 10.80 a
	Root (R)	CK_F_R	2.2536 ± 0.2644 a	86.75 ± 16.19 a
		Bn1_F_R	2.4455 ± 0.2195 a	95.41 ± 16.21 a
		SMC3_F_R	2.2425 ± 0.3436 a	99.80 ± 10.99 a
		SMC5_F_R	2.7450 ± 0.1152 a	92.30 ± 31.90 a
	Soil (S)	CK_F_S	3.4350 ± 0.2038 a	144.22 ± 11.28 a
		Bn1_F_S	3.6307 ± 0.1092 a	180.88 ± 14.16 a
		SMC3_F_S	3.5945 ± 0.1893 a	177.26 ± 15.80 a
		SMC5_F_S	3.5720 ± 0.1174 a	169.15 ± 30.81 a

Note: Data are presented as mean ± SD (n = 5). Different lowercase letters indicate significant differences among treatments according to one-way ANOVA followed by the Tukey–Kramer post hoc test ($p < 0.05$); identical letters indicate no significant differences. CK_F, pathogen-inoculated control without microbial treatment; Bn1_F, pathogen-inoculated plants treated with *Bacillus amyloliquefaciens* Bn1; SMC3_F and SMC5_F, pathogen-inoculated plants treated with the corresponding microbial consortium variants. The final letter in the sample code denotes the compartment: S, rhizosphere soil; R, roots; L, leaves.
