# Supplementary material for: Biocontrol Microbial Inoculants Suppress Fusarium oxysporum-Associated Disease Symptoms in Rice and Reshape Multicompartment Microbiomes
Source: Plants (Basel). 2026 Jun 26;15(13):1986. doi: 10.3390/plants15131986 (PMC13364377; doi:10.3390/plants15131986)
Supplement: Supplementary file 1 [file plants-15-01986-s001.zip › Figure S1.pdf]

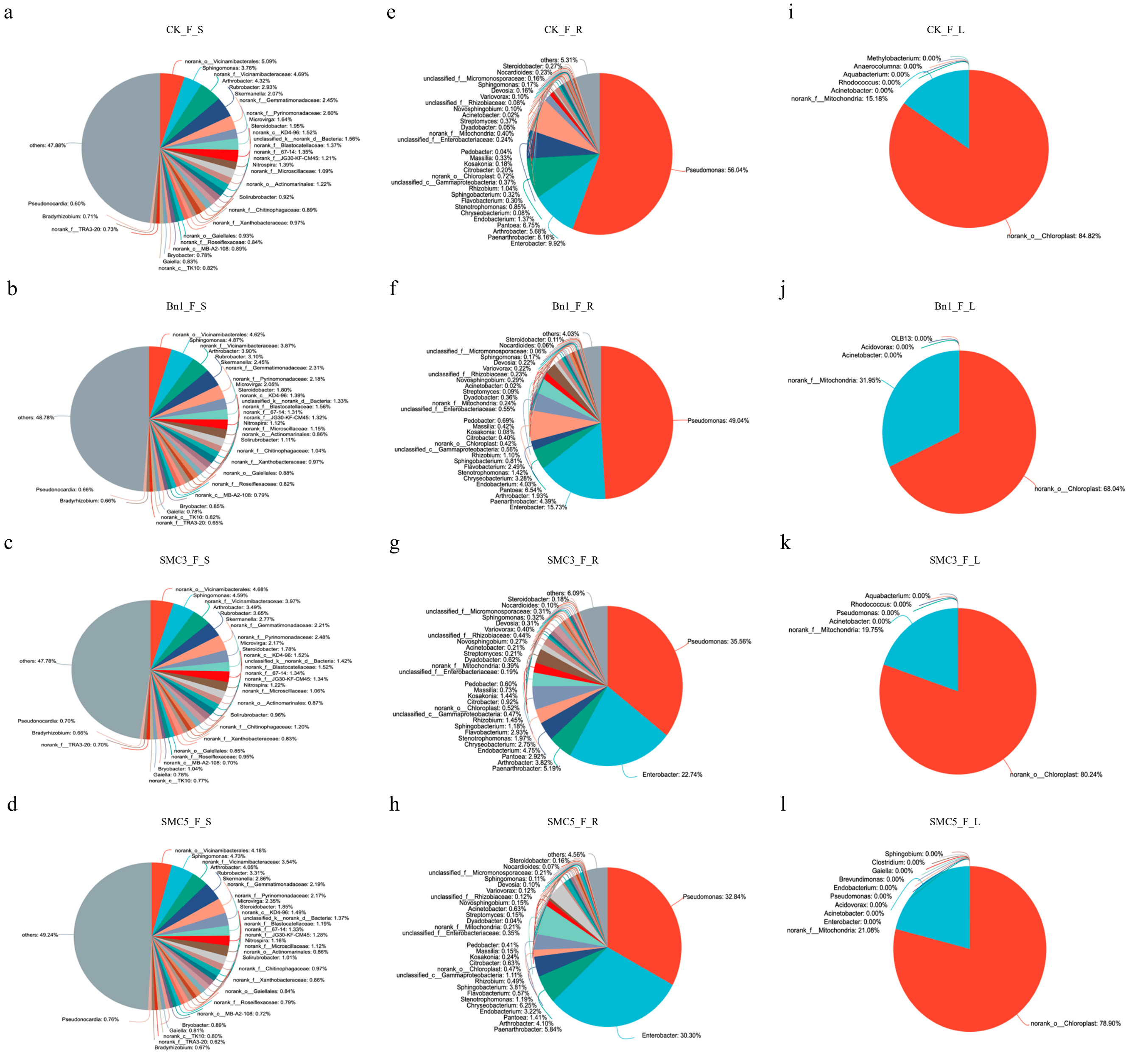

Figure S1. Relative abundance of dominant bacterial genera under different treatments: soil (a–d), roots (e–h), and leaves (i–l). Pie charts show the genus-level taxonomic structure of the bacterial community based on 16S rRNA sequencing data. The charts present genera that constitute the major proportion of the community; taxon names are shown according to the annotations in the graphs. Percentage values indicate relative abundance within each sample. CK_F, pathogen-inoculated control without microbial treatment; Bn1_F, pathogen-inoculated plants treated with *Bacillus amyloliquefaciens* Bn1; SMC3_F and SMC5_F, pathogen-inoculated plants treated with the corresponding microbial consortium variants. The final letter in the sample code denotes the compartment: S, rhizosphere soil; R, roots; L, leaves.
