# Supplementary material for: Biocontrol Microbial Inoculants Suppress Fusarium oxysporum-Associated Disease Symptoms in Rice and Reshape Multicompartment Microbiomes
Source: Plants (Basel). 2026 Jun 26;15(13):1986. doi: 10.3390/plants15131986 (PMC13364377; doi:10.3390/plants15131986)
Supplement: Supplementary file 1 [file plants-15-01986-s001.zip › Figure S2.pdf]

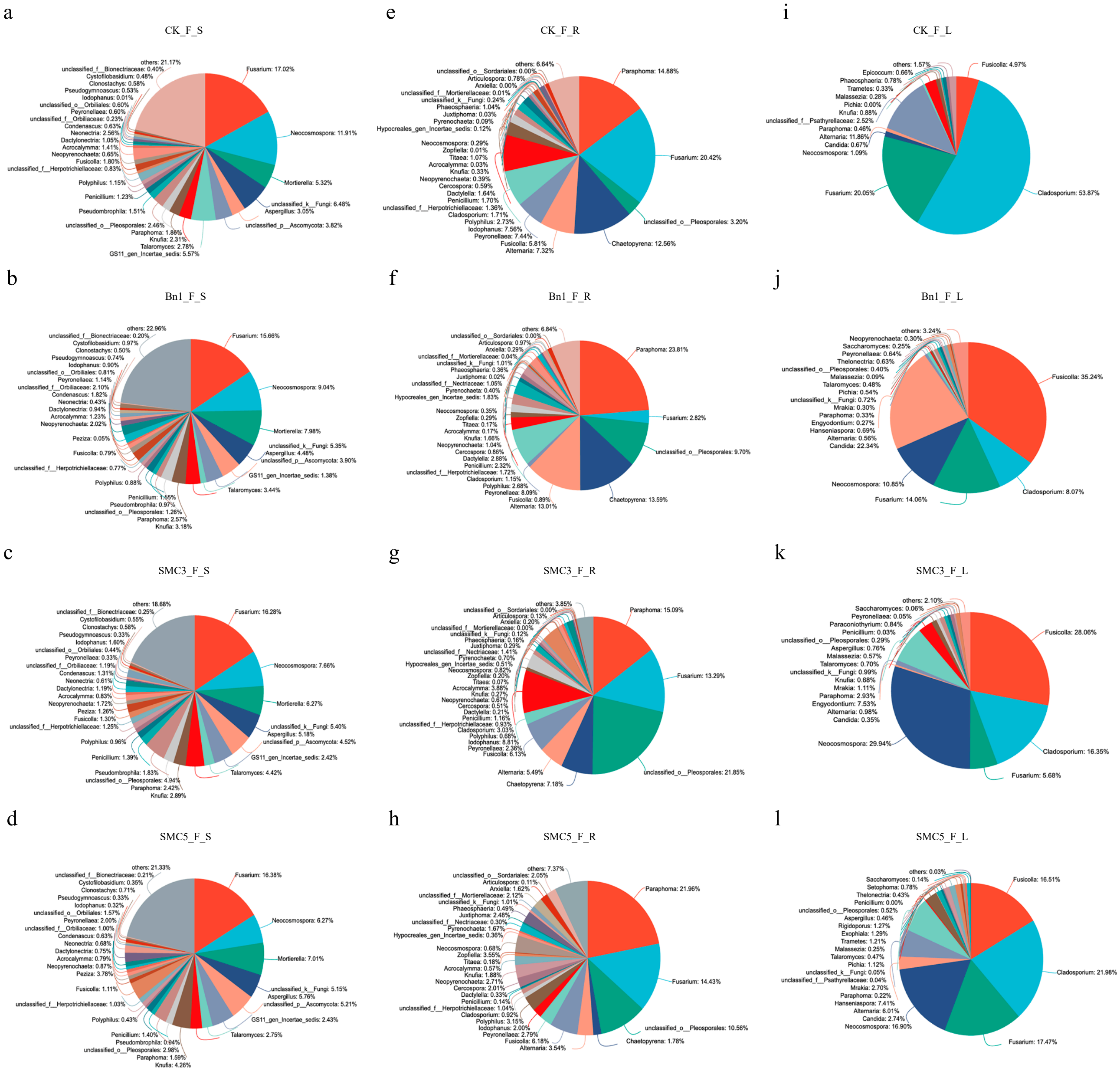

Figure S2. Relative abundance of dominant fungal genera under different treatments: soil (a–d), roots (e–h), and leaves (i–l). Pie charts show the genus-level taxonomic structure of the fungal community based on ITS sequencing data. The charts present genera that constitute the major proportion of the fungal profile. Percentage values indicate relative abundance within each sample. CK_F, pathogen-inoculated control without microbial treatment; Bn1_F, pathogen-inoculated plants treated with *Bacillus amyloliquefaciens* Bn1; SMC3_F and SMC5_F, pathogen-inoculated plants treated with the corresponding microbial consortium variants. The final letter in the sample code denotes the compartment: S, rhizosphere soil; R, roots; L, leaves.
