# Supplementary material for: Biocontrol Microbial Inoculants Suppress Fusarium oxysporum-Associated Disease Symptoms in Rice and Reshape Multicompartment Microbiomes
Source: Plants (Basel). 2026 Jun 26;15(13):1986. doi: 10.3390/plants15131986 (PMC13364377; doi:10.3390/plants15131986)
Supplement: Supplementary file 1 [file plants-15-01986-s001.zip › Table S1.pdf]

Table S1. Primary *in vitro* screening of antagonistic activity against *Fusarium oxysporum* F679

Variant	Mean ± SD, %	Selection
Nostoc sp. J-1	34.37 ± 1.05 i	–
Trichormus variabilis K-31	31.70 ± 0.95 i	–
Pseudomonas fluorescens Un1	41.77 ± 1.15 gh	–
Bacillus methylotrophicus Un2	45.43 ± 1.10 f	–
Bacillus amyloliquefaciens Bn1	58.70 ± 1.05 bc	+
J-1 + K-31	52.60 ± 0.95 d	+
Un1 + Un2 + Bn1	60.90 ± 1.10 b	+
Un1 + Un2	48.00 ± 1.10 ef	–
Un1 + Bn1	48.23 ± 1.05 ef	–
Un2 + Bn1	46.13 ± 1.00 ef	–
J-1 + Un1	44.97 ± 1.10 fg	–
J-1 + Un2	47.33 ± 1.05 ef	–
J-1 + Bn1	64.30 ± 0.95 a	+
K-31 + Un1	40.63 ± 1.15 h	–
K-31 + Un2	38.90 ± 1.05 h	–
K-31 + Bn1	48.97 ± 1.05 e	–
Un1 + J-1 + K-31	46.77 ± 1.15 ef	–
Un2 + J-1 + K-31	47.70 ± 1.00 ef	–
Bn1 + J-1 + K-31	49.20 ± 1.20 e	–
J-1 + Un1 + Un2 + Bn1	57.40 ± 1.05 c	+
K-31 + Un1 + Un2 + Bn1	49.10 ± 1.10 e	–
J-1 + K-31 + Un1 + Un2 + Bn1	55.80 ± 1.05 cd	+

Note: Data are mean ± SD (n = 3). Different lowercase letters indicate significant differences among treatments (one-way ANOVA with Tukey–Kramer post hoc, $p < 0.05$); identical letters indicate no differences. J-1 – *Nostoc* sp. J-1; K-31 – *Trichormus variabilis* K-31, Un1 – *Pseudomonas fluorescens* Un1; Un2 – *Bacillus methylotrophicus* Un2; Bn – *Bacillus amyloliquefaciens* Bn1.
